# Clinical outcomes of primary aldosteronism based on lateralization index and contralateral suppression index after adrenal venous sampling in real-world practice: a retrospective cohort study

**DOI:** 10.1186/s12902-020-00591-8

**Published:** 2020-07-29

**Authors:** Jeongmin Lee, Borami Kang, Jeonghoon Ha, Min-Hee Kim, Byungil Choi, Tae-Ho Hong, Moo IL Kang, Dong-Jun Lim

**Affiliations:** 1 Division of Endocrinology and Metabolism, Department of Internal Medicine, Eunpyeong St. Mary’s Hospital, College of Medicine, The Catholic University of Korea, Seoul, Republic of Korea; 2 Division of Endocrinology and Metabolism, Department of Internal Medicine, Seoul St. Mary’s Hospital, College of Medicine, The Catholic University of Korea, Seoul, Republic of Korea; 3 Department of Radiology, Seoul St. Mary’s Hospital, College of Medicine, The Catholic University of Korea, Seoul, Republic of Korea; 4 Department of Surgery, Seoul St. Mary’s Hospital, College of Medicine, The Catholic University of Korea, Seoul, Republic of Korea

**Keywords:** Hyperaldosteronism, Angiography, Selection, Index

## Abstract

**Background:**

We investigated whether the contralateral suppression index in aldosterone-producing adrenal adenoma could be used as a diagnostic criterion when catheterization in either right or left adrenal vein fails or when a discrepancy in the adrenal vein sampling (AVS) results and imaging findings occurs in the real-world practice.

**Methods:**

We retrospectively reviewed 48 patients who had resistant hypertension (HTN) or hypokalemia with a biochemical diagnosis of primary aldosteronism and who underwent AVS from January 2009 to June 2017 at a tertiary referral hospital. Selection index (SI), lateralization index (LI), and contralateral suppression index (CSI) were calculated based on AVS results and the final clinical outcomes were evaluated.

**Results:**

The catheterization of both adrenal veins was successful in 43 of 48 (89.6%) patients. The lateralization based only on LI was performed in 23 out of 43 (53.5%) patients. When CSI and LI were combined in decision making, the concordance between adrenal computed tomography scan and AVS for unilateral lesion improved from 59.3% (19/32) to 75.0% (24/32). CSI also correlated well with unilateral adrenal disease in the catheterization failure group. The final outcomes of HTN were better in the contralateral suppression group.

**Conclusion:**

CSI combined with LI could be a supplementary diagnostic tool in patients with non-lateralization or catheterization failure and predict the clinical outcomes of HTN in patients with primary aldosteronism.

## Background

Primary aldosteronism (PA) is the most common cause of secondary hypertension (HTN) affecting about 5 to 10% of hypertensive population [1, 2]. PA is caused by inappropriately high synthesis and secretion of aldosterone leading to high plasma sodium retention, suppression of plasma renin, and increased potassium excretion. These conditions lead to arterial HTN and hypokalemia. Prolonged PA increases the risk of target organ damage and cardiovascular morbidity and mortality [3]. Therefore, the early detection and appropriate treatment of PA is important for preventing progressive organ damage and cardiovascular complication. PA is commonly caused by a unilateral aldosterone-producing adrenal adenoma (APA), bilateral idiopathic adrenal hyperplasia (BAH) or rarely adrenal carcinoma. The classification of PA subtype is very crucial because unilateral APA is considered to be curable. The clinical practice guidelines of the Endocrine Society recommend adrenal computed tomography (CT) scan as the initial diagnostic imaging for classification of subtype after PA is screened with plasma aldosterone/renin ratio (ARR) [4]. Adrenal vein sampling (AVS) should be performed when surgery is decided to distinguish between unilateral and bilateral adrenal diseases. This procedure is invasive and difficult to perform; The success rate of both adrenal veins catheterization was presented in a various range (42–98%) [5]. Clinicians are often faced with conflicting results when conducting AVS and adrenal CT scan to differentiate between APA and BAH.

Recently, several studies used suppression of serum aldosterone levels in uninvolved contralateral adrenal as an optional criterion to confirm APA [6–8]. However, these data were investigated only in patients who underwent adrenalectomy immediately after AVS. The most crucial aim in the real world practice is to determine the appropriate treatment through early accurate diagnosis and to improve the patients’ outcome.

In this study, we aimed to investigate the result of the AVS performed in our institution and to evaluate the treatment outcomes based on these results. Moreover, we aimed to determine whether the contralateral suppression index could be used as a diagnostic criterion when catheterization fails or when a discrepancy in the AVS results and imaging findings occurs in the real-world practice.

## Methods

### Patient population and diagnostic methods

This study was a retrospective cohort study. We reviewed the records of 48 patients who were diagnosed with PA based on the results of the ARR over 20 and plasma aldosterone concentration (PAC) over 15 ng/dL [9] and those who underwent AVS between January 2009 and June 2017 at a tertiary referral hospital. Saline infusion test (SIT) is performed as a confirmatory test after withdrawal of antihypertensive agents which may influence plasma renin concentration for at least 2-to-4 weeks. The SIT started at 8: 00 A.M. Before the test, patients kept in the recumbent position for at least 2 h. After sampling for baseline PAC and renin with measurement of blood pressure (BP) with heart rate, 2 l of saline were infused over 4 h. PAC, renin, and BP with heart rate were measured after saline loading. PAC of 10 ng/dL in recumbent position or 6 ng/dL in seated position is considered as confirmative cut-off value [10]. All patients underwent adrenal CT scan imaging to check the morphological changes of both adrenal glands. All study participants received either medical or surgical treatments based on their integrated clinical information and AVS results. All patients were allowed for a 4- to-8 week visit to endocrinology clinics and were followed up at least 1 year after decisive AVS results.

### Hormone assay

PAC and cortisol concentrations were measured using radioimmunoassay (SPAC-S Aldosterone Kit; Fuji Rebio, Tokyo, Japan, cortisol kit; Beckman Coulter, Tokyo, Japan). The ARR was calculated by dividing the PAC by plasma renin activity (PRA; Fuji Rebio). If the ARR was greater than 20 and the plasma aldosterone level was greater than 15 ng/dL, the patient is diagnosed with PA [9]. As the detectable lower limit of plasma renin activity (PRA) was 0.10 ng/ml/hr. in our institution, PRA was calculated by assuming 0.10 ng/ml/hr. for the case reported as less than 0.10 ng/ml/hr.

### Adrenal vein sampling (AVS)

At least 8 weeks before AVS, antihypertensive agents such as angiotensin-converting enzyme inhibitors, angiotensin receptor blockers, diuretics, beta blockers, and direct vasodilators, which may influence plasma renin concentration, should be discontinued. The medication was switched to a non-dihydropyridine calcium channel blocker such as verapamil, which had no effect on plasma renin concentration. Hypokalemia was corrected with potassium supplement. AVS was performed by a single interventional radiologist with more than 10 years of experience in interventional vascular procedures. AVS was always performed in the morning to prevent false negative results due to diurnal fluctuation in adrenocorticotropic hormone (ACTH). 250 mcg of synthetic tetracosapeptide (cosyntropin) was loaded to uniformly stimulate aldosterone and cortisol secretion. A catheter was guided from the right femoral vein into the adrenal vein and infra renal inferior vena cava (IVC). After bilateral adrenal vein catheterization, blood samples were simultaneously obtained from IVC and bilateral adrenal veins. Repeated samples were obtained 10 and 20 min after a bolus dose of cosyntropin.

### Definitions of selectivity index, lateralization index, and contralateral suppression criteria

To evaluate the success of adrenal vein catheterization, selectivity index (SI) was defined as the ratio of cortisol concentration for each adrenal vein and IVC [11]:

$$ \mathrm{SI}=\kern0.5em \frac{\mathrm{Plasm}\mathrm{a}\ \mathrm{cortisol}\ \mathrm{concentration}\ \mathrm{of}\ \mathrm{adrenal}\ \mathrm{vein}}{\mathrm{Plasm}\ \mathrm{cortisol}\ \mathrm{concentration}\ \mathrm{of}\ \mathrm{IVC}} $$

Catheterization was considered successful if SI was at least threefolds higher at baseline and exceeded 5:1 at post-cosyntropin.

The lateralization index (LI) was defined as the aldosterone to cortisol (A/C) ratio on the dominant side with excess aldosterone secretion over A/C ratio on the non-dominant side [12]:

$$ \mathrm{LI}=\kern0.5em \frac{\left(\mathrm{Plasma}\ \mathrm{aldoterone}\ \mathrm{concentration}\right)/\left(\mathrm{plasma}\ \mathrm{cortisol}\ \mathrm{concentration}\right)\ \mathrm{of}\ \mathrm{dominant}\ \mathrm{adrenal}\ \mathrm{vein}}{\left(\mathrm{Plasma}\ \mathrm{aldoterone}\ \mathrm{concentration}\ \right)/\left(\mathrm{plasma}\ \mathrm{cortisol}\ \mathrm{concentration}\right)\mathrm{of}\ \mathrm{non}\hbox{-} \mathrm{dominant}\ \mathrm{adrenal}\ \mathrm{vein}} $$

The A/C ratio of the dominant adrenal vein was more than twofold higher at baseline and fourfold higher at post-cosyntropin in lateralization. If the A/C ratio of the dominant side was less than 3 times the A/C ratio of the non-dominant side at post-cosyntropin in lateralization, BAH was suggested [12].

The contralateral adrenal zona glomerulosa be absolutely suppressed in unilateral PA. Therefore, contralateral suppression was defined based on the assumption that the uninvolved adrenal vein aldosterone level might be less than the serum normal aldosterone level, which was measured in the IVC. Contralateral suppression index (CSI) was calculated as follows:

$$ \mathrm{CSI}=\kern0.5em \frac{\left(\mathrm{Plasma}\ \mathrm{aldoterone}\ \mathrm{concentration}\right)/\left(\mathrm{plasma}\ \mathrm{cortisol}\ \mathrm{concentraion}\right)\ \mathrm{of}\ \mathrm{the}\ \mathrm{nondominant}\ \mathrm{adrenal}\ \mathrm{vein}}{\left(\mathrm{Plasma}\ \mathrm{aldoterone}\ \mathrm{concentration}\right)/\left(\mathrm{plasma}\ \mathrm{cortisol}\ \mathrm{concentraion}\right)\mathrm{of}\ \mathrm{the}\ \mathrm{IVC}} $$

Contralateral suppression was confirmed if the uninvolved adrenal A/C ratio was less than 1.0 compared with A/C ratio of the IVC [7].

### Definition of clinical outcome after adrenalectomy or medical therapy

The clinical outcomes of PA were evaluated at least 1 year after decision making post AVS. Due to limited data availability from this retrospective study, the final clinical outcomes only consist of the clinical components including BP rather than biochemical parameters such as PAC, ARR, and potassium. The clinical outcomes were defined from the Primary Aldosteronism Surgical Outcome study [13]. Initial BP has been measured in outpatient clinic setting with seated position by a standard mercury sphygmomanometer or automated oscillometric devices. Higher BP was considered as initial BP if there was measurement of both arms. If the multiple measurement of BP was obtained, mean BP was calculated. The final SBP and DBP were measured three times on other day visit and the average value was computed. To classify the clinical success, antihypertensive agents were expressed as defined daily dosage (DDD) of initial diagnosis and of post-therapeutic decision. DDD is assumed average maintenance dose per day and is calculated according to the definition by World Health Organization [14]. The DDDs assigned for multiple drugs or combination were based on the main principle of counting the cumulative unit of 1 day. A change in the DDD was defined as (pre-therapeutic DDD-post-therapeutic DDD)/ pre-therapeutic DDD × 100. Same DDD was defined as a change of less than 50% between initial DDD and post-therapeutic DDD. Reduced or increased DDD was defined as a change greater than 50%. After surgical or medical therapy, complete clinical success was indicated by a normal BP without antihypertensive agents, as noted in the European Society of Hypertension guidelines [15] for outpatient setting [systolic BP (SBP) less than 140 mmHg or a diastolic BP (DBP) less than 90 mmHg]. Partial clinical success was defined as reduction in BP levels with same or less DDD. Absent clinical success was defined as a having a BP higher than the baseline at the time of initial diagnosis or same BP even after using a same or higher DDD.

### Statistical analysis

All statistical analyses were performed using SPSS software (version 14; SPSS Inc., Chicago, IL, USA). The distributions of all continuous variables were examined using the Shapiro-Wilk test. The non-normally distributed variables were reported as median and range. Frequencies and percentages were used for all categorical variables (gender, HTN history, and CT findings). The characteristics of lateralized group and non-lateralized group were compared using the independent t-test (continuous variables) or the chi-square and Fisher’s exact test (categorical variables). A two-tailed *p*-value of < 0.05 was considered significant.

## Results

### Baseline characteristics of the study subjects

A total of 43 patients were included in the study while five patients were excluded due to failure to achieve a successful catheterization of the unilateral or bilateral adrenal veins based on the SI [89.6%(43/48) success rate in our institution]. Before initial ARR test for screening PA, 20% of the patients were taking beta-blockers, 39% were taking calcium channel blockers, 6% were taking angiotensin receptor blockers or angiotensin-converting enzyme inhibitors, and 4.7% were taking diuretics (data not shown). Adrenal CT scan was performed in all patients; but five (11.6%) patients showed no definite abnormalities of the adrenal gland on CT scan. Clinical characteristics of enrolled patients were described in detail in Table 1.

**Table 1:** Baseline clinical characteristics of 43 patients with primary aldosteronism

Clinical parameters	***N*** = 43
Age (range), (years)	48 (19–80)
Gender (Male/Female) (%)	17/26 (65.4/34.6)
Patients with hypertension (%)	40 (93.0)
Numbers of antihypertensives	3 (0–5)
Initial DDD	2.2 (0.0–4.8)
SBP (mmHg)	150 (110–204)
DBP (mmHg)	90 (63–160)
K (mEq/L)	3.2 (1.6–5.5)
PAC (ng/dL)	39.6 (27.0–227.2)
Plasma renin activity (ng/ml/h)	0.10 (0.10–2.4)
ARR	304.4 (34.5–1535.0)
Adrenal CT finding	
Unilateral adrenal mass (%)	32/43 (74.4)
Bilateral hyperplasia or bilateral adenoma (%)	6/43 (14.0)
No definite lesion (%)	5/43 (11.6)
Size of adrenal mass (cm)	1.5 (0.0–7.3)
Follow-up duration (months)	30.0 (12.6–86.0)

Nonparametrically distributed data presented as median with range

Frequencies and percentages were used for all categorical variables such as gender and CT findings

*DDD* defined daily dose; SBP, systolic blood pressure, *DBP* diastolic blood pressure, *K* potassium, *PAC* plasma aldosterone concentration, *ARR* plasma aldosterone concentration/renin activity ratio, *CT* computed tomography

### Decision making based on AVS results with LI

**Fig. 1:**
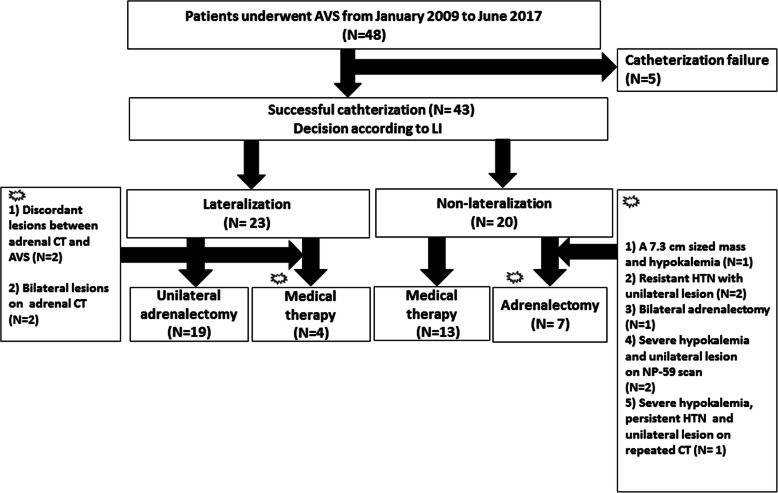
Decision making based on AVS results with LI

Among 43 patients, 23 (53.5%) presented unilateral adrenal disease and 20 (46.5%) had bilateral adrenal disease based on AVS results with LI. Figure 1 demonstrates the decision making process for adrenalectomy or medical therapy based on the LI from AVS. Overall, 19 out of 23 patients who showed lateralization underwent unilateral adrenalectomy (the lowest leftmost box in Fig. 1). Among these 19 patients, 14 with concordant adrenal CT scan and AVS results had immediate unilateral adrenalectomy after the first AVS. Additional adrenalectomy was performed in the remaining two patients during the follow-up period: One patient with uncontrolled blood pressure and increased size of adrenal adenoma and the other with repeated severe hypokalemia. Another three patients with unilateral lesion on adrenal CT underwent adrenalectomy with lateralization on repeat AVS results.

The remaining four patients with discordant adrenal CT scan and AVS results were treated with medications including aldosterone antagonist and antihypertensive agents during the study (lowest second left box in Fig. 1); two patients showed discordant unilateral lesions based on the results of the adrenal CT scan and AVS. These patients refused adrenalectomy. The remaining two patients showed bilateral lesions on the adrenal CT scan but showed lateralization to one side on AVS. They were kept on medical therapy and were on short-term follow-up.

A total of 7 patients without lateralization on initial AVS underwent adrenalectomy (the lowest rightmost box in Fig. 1). Four out of ten patients underwent adrenalectomy immediately after AVS. One patient with a 7.3-cm adrenal mass and hypokalemia underwent unilateral adrenalectomy despite non-lateralization. Two patients with resistant HTN and unilateral lesion on adrenal CT scan, taking four types of antihypertensive agents, underwent unilateral adrenalectomy of the dominant adrenal gland according to clinician’s decision. The final patient underwent bilateral adrenalectomy based on the results of the adrenal CT scan and AVS, which were suspicious for bilateral hypersecretion and persistent HTN despite administration of a combination of antihypertensive agents.

The remaining three patients were kept on antihypertensive treatment after initial AVS (the lowest rightmost box in Fig. 1). Two patients developed severe hypokalemia (1.6 mEq/L and 1.8 mEq/L, respectively) 9 months and 1 year, respectively, after initial AVS. Hence, the physician performed adrenal 131I-iodocholesterol (NP-59) scintigraphy to identify active lesions within both adrenal glands, without repeated AVS due to patients’ refusal. NP-59 scan showed active unilateral lesions, which were concordant with the adrenal CT scan findings. Thus, unilateral adrenalectomy was performed. Lastly, one patient had no definite lesion on CT scan at the time of AVS and showed non-lateralization on AVS. On repeat adrenal CT scan, which was performed because of recurrent hypokalemia and uncontrolled blood pressure, a 0.7-cm unilateral nodular lesion was newly detected 12 months after initial AVS and adrenalectomy was performed. AVS-guided therapeutic plan was decided in 32 of 43 (74.4%) (adrenalectomy 19 + medical therapy 13).

### Changes in decision making based on AVS results with LI and CSI

**Fig. 2:**
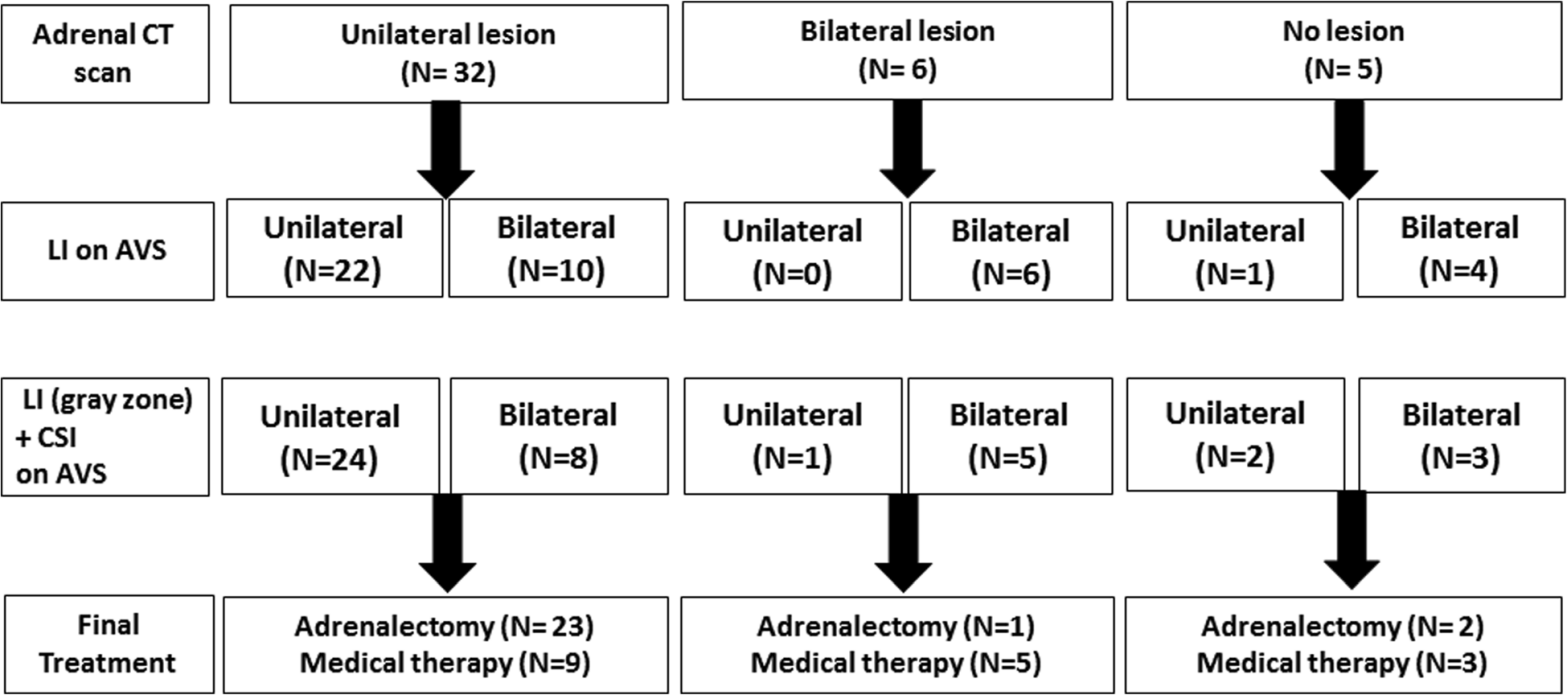
Final treatment status according to adrenal CT scan and AVS results with LI only or with LI and CSI

Figure 2 shows a flow chart of the changes in the decision-making process based on the adrenal CT scan and AVS results with LI and CSI.

We applied CSI [6] to study participants by retrospective analysis (Fig. 2, 3rd row). Among 32 patients with unilateral lesion on adrenal CT scan, 24 showed definite contralateral suppression regardless of lateralization and 23 of those patients underwent adrenalectomy, indicating additional two patients (22➙ 24) were re-categorized from bilateral to unilateral lesion, addition of CSI.

One out of six patients with bilateral lesions on adrenal CT scan showed lateralization based on the AVS results with LI and CSI. This patient finally underwent adrenalectomy based on the results of the CSI and due to severe hypokalemia and unilateral lesion as shown on repeated adrenal CT scan.

Among five patients who showed no definite lesion on initial adrenal CT scan (Fig. 2, upper rightmost box), two had unilateral lesions based on the AVS results with LI and CSI. During follow-up, these two patients suffered from severe hypokalemia and showed active unilateral lesion on adrenal NP scan; thus, unilateral adrenalectomy was performed.

The correlation of adrenal CT scan was higher in LI and CSI together than in LI alone. When CSI and LI considered together, the diagnostic performance for differentiating unilateral APA was improved in sensitivity, negative predictive value, and accuracy compared to applying only LI (Table 2 and Table 3).

**Table 2:** Results of correlation of adrenal CT scan, LI and CSI in all 43 study subjects

Lesion on CT scan	LI		LI and CSI	
	Lateralization (*n* = 23)	Non-lateralization (*n* = 20)	Lateralization (*n* = 27)	Non-lateralization (*n* = 16)
Unilateral lesion (*n* = 32)	22	10	24	8
Bilateral or no lesion (*n* = 11)	1	10	3	8

*CT* computed tomography, *LI* lateralization index, *CSI* contralateral suppression index

**Table 3:** Diagnostic performance of LI, LI and CSI in all 43 study subjects

Diagnostic tool	Diagnostic value				
	Sensitivity (%)	Specificity (%)	PPV (%)	NPV (%)	Accuracy (%)
LI	59.8 *	77.8 *	96.0	43.5	67.4
LI and CSI	75.0 *	72.4 *	88.9	50.0	74.4

* *P <* 0.05, derived from a McNemar test

*PPV* positive predective vale, *NPV* negative predictive vale

### Final clinical outcomes of study subjects

Table 4 demonstrates the final clinical outcomes of the study participants. In the lateralization group (*n* = 23), the median follow-up duration was 36.5 months (range: 12.6–86.0 months). Among them, 19 patients underwent unilateral adrenalectomy and four were kept under medical therapy. There were no significant differences in initial DDD between adrenalectomy and medical therapy. The post-therapeutic DDD was lower in patients with adrenalectomy than in patients with medical therapy (0.5 unit vs. 2.3, *p* = 0.003), implying adrenalectomy was more effective in reducing daily maintenance dose of antihypertensives. After adrenalectomy, surgical pathology confirmed adrenal cortical adenoma in 17 patients and adrenal cortical hyperplasia in two patients. Among patients who underwent adrenalectomy, 10 (52.6%) achieved clinical success while eight (42.1%) showed partial clinical success, even when the post-therapeutic DDD was reduced. However, one (5.3%) patient who underwent adrenalectomy and showed adrenal hyperplasia in postoperative pathology failed to achieve clinical success. Among four patients who received medical therapy, two (50%) maintained partial clinical success. The other two (50%) patients with bilateral lesions on CT scan were treated by a higher dose of spironolactone (up to 100 mg) but still showed uncontrolled blood pressure.

**Table 4:** Clinical outcomes according to treatment based on LI in AVS results

Parameters	Lateralization (***N*** = 23)		***P*** value	Non-lateralization (***N*** = 20)		***P*** value
Clinical outcomes according to treatment	Adrenalectomy *N* = 19 (82.6%)	Medical therapy *N* = 4 (17.3%)		Adrenalectomy *N* = 7 (35.0%)	Medical therapy *N* = 13 (65.0%)	
Initial DDD	2.6 (1.5–4.4)	2.31 (0.5–4.0)	0.225	1.9 (0.0–3.6)	1.7 (0.0–4.8)	0.814
Post-therapeutic DDD	0.5 (0.0–2.0)	2.3 (1.1–4.6)	0.003	0.8 (0.0–3.1)	1.3 (0.0–4.6)	0.157
Change in DDD (%)	- 70.0 (− 100–0)	+ 36 (− 37.8 − + 120)	0.001	- 40.3 (− 100 − + 34)	- 15.6 (− 3.33 − + 230)	0.018
Clinical success			0.000			0.303
Complete success	10	0		4	0	
Partial success	8	2		1	10	
Absent success	1	2		2	3	

*DDD* defined daily dose

Change in DDD (%), pre-therapeutic DDD-post-therapeutic DDD)/pre-therapeutic DDD ×100.

In the non-lateralization group who had a median follow-up of 29.5 months (range: 8.6–47.8 months), 7 patients underwent adrenalectomy; surgical pathology presented adrenal adenoma in six patients and medullary diffuse hyperplasia in one patient. There were no significant differences in initial DDD and in post-therapeutic DDD between adrenalectomy and medical therapy. The change in DDD % was significantly higher in adrenalectomized patients. Among the 7 patients who underwent adrenalectomy, four achieved complete clinical successes without medical therapy, one achieved partial clinical success with medical therapy and two patients failed to achieve clinical success. About 13 patients who received medical therapy without adrenalectomy rarely achieved complete clinical success. Ten showed partial clinical success the remaining three patients failed clinical success.

We compared the outcomes between patients with contralateral suppression and patients without contralateral suppression in the non-lateralization group (Table 5). The rate of complete clinical success for HTN after adrenalectomy was superior in patients with contralateral suppression compared with those without suppression [33.3% (3 of 9) vs. 9.1% (1 of 11)]. The overall clinical success rate (complete clinical success and partial clinical success) for HTN was not different between contralateral suppression and non-contralateral suppression [ 88.9% (8/9) vs. 81.8% (8/11) *p* = 0.052]. Absent clinical success showed a higher trend in patients without contralateral suppression, but no significant difference was observed (*p* = 0.052), probably due to small number.

**Table 5:** Baseline characteristics and outcome in non-lateralized group according to contralateral suppression

	Non-lateralized group (*N* = 20)		*p*-value
	Contralateral suppression (*N* = 9)	Non-contralateral suppression (*N* = 11)	
Initial DDD	1.7 (0.5–4.8)	1.6 (0.0–3.7)	0.741
Post DDD	0.8 (0.0–2.3)	1.1 (0.0–4.6)	0.194
Change in DDD %	−34.0 (−100 − + 135)	−16.1 (0.0 − + 230)	0.046
Lateralization index, baseline	2.1 (1.3–2.6)	1.9 (1.0–2.4)	0.823
Lateralization index, post ACTH	2.0 (1.0–2.4)	1.6 (1.0–2.8)	0.431
Contralateral suppression index*	0.5 (0.3–0.9)	2.4 (1.3–5.1)	0.035
PAC (ng/dL)	44.1 (25.4–227.2)	36.5 (21.4–119.2)	0.758
Plasma renin activity (ng/dL)	0.38 (0.10–1.43)	0.16 (0.10–0.39))	0.014
ARR	307.4 (38.4–1192.0)	404.21 (62.1–1263.1)	0.933
Post-therapeutic SBP (mmHg)	125.8 (115–148)	128.2 (107–160)	0.991
Post-therapeutic DBP (mmHg)	82.4 (70–103)	89.8 (60–120)	0.896
Post PAC (ng/dL)	20.0 (5.4–40.5)	19.0 (5.3–48.6)	0.654
Post Plasma renin activity (ng/ml/h)	1.4 (0.27–3.50)	4.51 (0.10–21.25)	0.076
Post ARR	22.1 (1.6.4)	40.7 (4.5–131.8)	0.135
Clinical outcomes			
Complete clinical success	3/9 (33.3)	1/11 (9.1)	0.001
Partial clinical success	5/9 (55.6)	7/11 (63.6)	0.699
Absent clinical success	1/9 (11.1)	3/11 (27.2)	0.052

*The contralateral suppression index was defined as uninvolved adrenal A/C ratio compared to A/C ratio of IVC post than 1.0

*DDD* defined daily dose, *LI* lateralization index, *PAC* plasma aldosterone concentration, *ARR* plasma aldosterone concentration/renin activity, *SBP* systolic blood pressure, *DBP* diastolic blood pressure

### Decision making and clinical outcome of five patients with catheterization failure

We analyzed five patients who had a failed catheterization. The baseline characteristics are summarized in Table 6. Unilateral left catheterization was successful in three out of five patients (no. 1, 2, and 3) and bilateral catheterization failure occurred in two patients (no. 4 and 5). Unilateral failure was limited only to cases of unsuccessful right side catheterization. If CSI was applied to these study subjects, three patients with a CSI of less than 1.0 could have been categorized as having a unilateral disease (no. 1, 2, and 3). Among these three patients, two (no. 1 and 3) had persistent HTN even with the use of multiple antihypertensive agents and showed definite unilateral adrenal lesion on adrenal CT scan. Both patients underwent adrenalectomy during follow-up and achieved complete clinical success. The remaining (no. 2) patient developed severe hypokalemia (< 2 mEq/L) with uncontrolled HTN despite taking 100 mg of spironolactone. Without repeat AVS, NP-59 scan showed definite activity in the right adrenal gland, which was consistent with the findings of the adrenal CT scan. This patient underwent right adrenalectomy and finally achieved partial clinical success. Surgical pathology confirmed adrenal cortical adenoma in these patients (no. 1, 2, and 3). Two patients (no. 4 and 5) with bilateral catheterization failure were kept under therapy. The SBP and DBP of these patients remained unchanged without increasing the dosage of their medication.

**Table 6:** Initial clinical characteristics of patients with cathterization failure

No	Age, range (years)	SBP/DBP (mmHg)	K (mEq/L)	ARR	Location on adrenal CT	SI* Rt./Lt	CSI**	Adrenalectomy
1	40–49	150/90	3.0	264.9	Rt.	1.13/44.44	0.85	Yes
2	20–29	161/108	1.9	498.6	Bilateral	1.37/30.47	0.65	Yes
3	60–69	130/72	3.0	413.9	Rt.	1.0/17.90	0.22	Yes
4	50–59	158/107	4.2	34.6	Bilateral	1.17/1.13	1.19	No
5	80–89	150/80	2.2	82.8	Bilateral	1.18/1.04	1.14	No

*SBP* systolic blood pressure, *DBP* diastolic blood pressure, *K* potassium, *ARR* plasma aldosterone concentration/renin activity ratio, *CT*, computed tomography

*SI, Selectivity index: adrenal vein to IVC cortisol concentration ratio

**CSI, Contralateral suppression index: uninvolved adrenal A/C ratio compared to A/C ratio of IVC

## Discussion

As more than 42% of APA patients with HTN and more than 95% with hypokalemia can be cured after adrenalectomy [16], recognizing the PA subtype is very crucial to decision making and improvement of outcomes. Usually, adrenal CT scan is needed for localization after biochemical diagnosis of PA. In several studies, the accuracy of adrenal CT scan findings in APA patients was reported to be less than 50% [9, 17]. According to a systemic review of 950 patients [1], if the adrenal CT finding was only regarded, unnecessary adrenalectomy would be performed in 19% and opposite adrenalectomy in 4%. Several studies reported that AVS is a reliable diagnostic tool for localization [11, 18–20]. Moreover, one study insisted that routine AVS should be performed because 25% of APA patients presented a negative CT finding [21].

However, AVS is a technically difficult method and success rates are often low [22]. Consensus for interpreting AVS results vary from center to center. In our study, 53.5% (23 of 43) of patients showed concordant adrenal CT scan and AVS results. For the remaining 46.5% of the patients, it was difficult for the clinicians to decide which treatment should be used based on the adrenal CT and AVS results with LI.

Several studies investigated the utility of CSI for differentiating APA from BAH and for predicting the outcomes of patients with HTN and hypokalemia [6, 7, 23–26]. LI with cosyntropin infusion greater than 4 is definitively diagnostic of APA and LI less than 2 [27] is diagnostic of BAH. The multicenter study concluded that CSI should not be required for all patients with LI greater than 4. However, the gray zone of LI with cosyntropin infusion (between 3 and 4) [19, 28] is difficult to confirm the localization. Contralateral suppression showed a clue for the source of aldosterone over-production and hypersecretion [29]. In this case, CSI could be considered for decision making. However, one study [12] suggested that the prediction of APA cannot be confirmed only by CSI because 30% of BAH patients showed contralateral suppression indicated by a CSI less than 1. Several studies reported that left sided adenoma was significantly larger and prominent than right adenoma among bilateral adenomas [30–33].

One previous study [7] and ours definitely showed LI with CSI was superior to LI only to confirm the lateralization and to make a clinical decision. The final concordant rate between adrenal CT scan and AVS results based on LI was 43.8% (14/32), whereas that between adrenal CT scan and AVS based on LI with CSI was 71.9% (23/32). All these patients showed LI between 2 and 4. Therefore, the CSI should be considered in bilateral lesions where CT scan and AVS results do not match properly. CSI should be used in decision making with unilateral adrenal lesion for surgery.

Patients with contralateral suppression were likely to have unilateral lesion and show lower postoperative blood pressures and higher biochemical evidence of cure [6, 7, 25, 34]. Contralateral adrenal gland which is not suppressed could have residual aldosterone production and HTN would be maintained even after adrenalectomy [35]. These studies were limited only to patients who underwent adrenalectomy after confirmation of unilateral lesion. Unlike previous studies, we evaluated the outcome of all patients regardless of adrenalectomy. In the non-lateralization group, the rate of complete clinical success for HTN was higher in patients with contralateral suppression than in those without contralateral suppression. The contralateral suppression definitely showed trends toward higher rate of clinical success, suggesting that it is not proper to recommend adrenalectomy without contralateral suppression in the non-lateralization group. However, as the clinical success was evaluated only by blood pressure, not by aldosterone level or ARR because of the retrospective design of the study, whether CSI can predict better clinical success remained uncertain.

The physician decides the type of medical therapy for non-lateralized PA. In the current study, the partial clinical success with medical therapy was 43.5% (10 out of 23) in the non-lateralization group. Approximately 13% (3 of 23) of patients were not able to achieve clinical success. This result is similar to that reported in the previous study [36] which investigated that only 44% of medical therapy patients with non-lateralization reached a blood pressure level of < 140/90 using the same or a lower dose of medication, which is equivalent to partial clinical success in our study.

In the additional analysis, we applied CSI to five catheterization failure patients. The recent studies [26, 37] showed that if the catheterization failure occurred in the right vein, left CSI could be useful for localization. Our results were consistent with previous studies and three patients showed contralateral suppression on the left side, indicating a right-side disease. Therefore, these patients were appropriate surgical targets based on CSI from AVS. CSI could be a supplementary tool to interpret incomplete AVS results in case of one side catheterization failure.

The present study has several limitations. First, this study was based on cross-sectional and retrospective data and it might be associated with a selection bias. Second, because of the small sample size, the results might show insignificant differences in several important clinical factors including postoperative blood pressure changes. Third, complete clinical success was defined only as keeping blood pressure within the normal range without antihypertensive agents, not by biochemical cure of PAC and ARR, due to limited data. Because there was sparse study of predefined clinical success in medical therapy, an evaluation of clinical outcomes in medical therapy was limited.

## Conclusions

CSI correlates well in unilateral disease if LI is in the gray zone and could be useful as a supplementary predictor of lateralization. Moreover, CSI could be used in cases of catheterization failure in the unilateral vein of dominant lesion. Contralateral suppression presented a higher rate of complete clinical success without antihypertensive agents. However, a long-term follow-up study with a large population is required to validate these results.

## Data Availability

All the data generated and/or analyzed during the current study are included in this article and are available from the corresponding author on reasonable request.

## Abbreviations

ACTH: Adrenocorticotropic hormone
APA: Aldosterone-producing adrenal adenoma
ARR: Plasma aldosterone/renin ratio
AVS: Adrenal vein sampling
BAH: Bilateral idiopathic adrenal hyperplasia
BP: Blood pressure
CSI: Contralateral suppression index
CT: Computed tomography
DBP: Diastolic blood pressure
DDD: Defined daily dosage
HTN: Hypertension
IVC: Inferior vena cava
LI: Lateralization index
PA: Primary aldosteronism
PAC: Plasma aldosterone concentration
SBP: Systolic blood pressure
SIT: Saline infusion test
SI: Selectivity index
